# Plasticity of visual attention in Isha yoga meditation practitioners before and after a 3-month retreat

**DOI:** 10.3389/fpsyg.2013.00914

**Published:** 2013-12-12

**Authors:** Claire Braboszcz, B. Rael Cahn, Bhavani Balakrishnan, Raj K. Maturi, Romain Grandchamp, Arnaud Delorme

**Affiliations:** ^1^Centre de Recherche Cerveau et Cognition, UPS, Université de ToulouseToulouse, France; ^2^CERCO, CNRS UMR5549Toulouse, France; ^3^Department of Psychiatry and Human Behaviour, UC IrvineOrange, CA, USA; ^4^Isha Yoga Centre, Isha FoundationCoimbatore, India; ^5^Department of Ophthalmology, Volunteer, Indiana University School of Medicine Shareholder, Midwest Eye InstituteIndianapolis, IN, USA; ^6^SCCN, INC, UCSDLa Jolla CA, USA

**Keywords:** meditation, attention, Stroop task, attentional blink, global-local task

## Abstract

Meditation has lately received considerable interest from cognitive neuroscience. Studies suggest that daily meditation leads to long lasting attentional and neuronal plasticity. We present changes related to the attentional systems before and after a 3 month intensive meditation retreat. We used three behavioral psychophysical tests - a Stroop task, an attentional blink task, and a global-local letter task-to assess the effect of Isha yoga meditation on attentional resource allocation. 82 Isha yoga practitioners were tested at the beginning and at the end of the retreat. Our results showed an increase in correct responses specific to incongruent stimuli in the Stroop task. Congruently, a positive correlation between previous meditation experience and accuracy to incongruent Stroop stimuli was also observed at baseline. We also observed a reduction of the attentional blink. Unexpectedly, a negative correlation between previous meditation experience and attentional blink performance at baseline was observed. Regarding spatial attention orientation as assessed using the global-local letter task, participants showed a bias toward local processing. Only slight differences in performance were found pre- vs. post- meditation retreat. Biasing toward the local stimuli in the global-local task and negative correlation of previous meditation experience with attentional blink performance is consistent with Isha practices being focused-attention practices. Given the relatively small effect sizes and the absence of a control group, our results do not allow clear support nor rejection of the hypothesis of meditation-driven neuronal plasticity in the attentional system for Isha yoga practice.

## 1. Introduction

Behavioral results show that meditation practice increases performance in attentional tasks suggesting improved allocation of attentional resources, enhanced sustained attention skills, faster re-allocation of attentional resources, improved cognitive flexibility, and decreases in automatic responding (Valentine and Sweet, 1999; Carter et al., 2005; Cahn and Polich, 2006; Slagter et al., 2007; Hodgins and Adair, 2010). These changes are most likely linked to structural, anatomical and functional changes observed in meditators compared to control populations (Hoelzel et al., 2011; Luders et al., 2011, 2012; Kang et al., 2013). Lutz et al. (2008) have outlined a theoretical framework for the study of meditation according to which meditation practice involve at least three attention regulation subsystems. Meditation may help train selective attention, which pertains to the selection of information from the flow of sensory inputs. Meditation also requires sustained attention for continuous monitoring of the object of meditation. Finally while meditating, one often has to redirect attention from a source of distraction toward the intended object of meditation. These transient attentional shifts involve executive attention, that is the monitoring and resolution of conflict among thoughts, feelings and mental plans. Although useful to define the scope of our study and interpret our results this taxonomy is likely an oversimplification as the different attentional systems are most likely strongly intermingled. Meditation practices are usually classified depending on the attentional engagement they require, along a continuum from focused attention to mindfulness practices (Cahn and Polich, 2006; Lutz et al., 2007, 2008). Focused attention meditation techniques involve sustained attention on a selected internal or external object of awareness, whereas mindfulness meditation, also known as open-monitoring or open-awareness meditation, involves adopting an attentive and non-elaborative cognitive stance toward anything that may occur from moment-to-moment in the mental field of experience. Recently, the “automatic self transcending” category has been proposed to denote practices marked by the absence of individual control or effort during meditation thus leading to a transcending of the sense of self as a separate choosing agent, a characteristic of meditation practice that likely is related to the notion of non-dual awareness and varies from one practice to another in ways not fully characterized as of yet (Travis and Shear, 2010; Josipovic, 2013).

Isha Yoga, the meditation tradition studied in this article, includes many practices and, specifically during the 3 month retreat studied in this article, three primary practices were done daily which may be categorized into each of these three categories. *Lingasanchalana* meditation, in which the practitioner is seated and focused on one point, with eyes either opened or closed, is primarily a focused attention practice. *Samyama* meditation is a seated meditation involving the instruction to pay attention to the breath with eyes fully or partially open and passively observing thoughts in addition to watching the breath. This is a practice with a focused attention basis in which the instruction is to continually attend to the breath, although there is some open-monitoring/mindfulness component to this practice as well. *Shoonya* meditation is typically done after a set of physical postures and breathing exercises and involves sitting with eyes closed and engaging in a process of conscious non-doing that purportedly creates a distance between ones self and ones body and mind. This practice could be considered a form of open-awareness practice with self-transcending occurring through a non-doing aspect or alternatively conceived as a focused practice in that the explicit focus is on “non-doing”—as soon as one notices mental content arising in awareness the injunction is to attempt to reinstate a “non-doing”/nothingness experience and to use a mantra if necessary to do so. Alternatively, within the Isha yoga tradition, this practice is spoken of as a self-transcending practice and may be in line with the proposed self-transcending style of practice. The nuances of these practices and the best and most inclusive and accurate classification system for meditative practices is beyond the scope of this article. In addition to these three meditation techniques, Isha Yoga also includes practice of diverse yoga postures, breathing and physical exercise as well as chanting.

To assess the extent to which Isha Yoga meditation affects the processing of visual information, we used three attentional tasks corresponding to three different attentional characteristics: the attentional blink task (temporal attention), the Stroop task (conflict monitoring/executive attention and automated responding), and a global-local task (spatial attention to global vs. local aspects of the visual field). The subjects were tested at the beginning and end of a 3-month full time Isha Yoga meditation retreat.

In the attentional blink task (Raymond et al., 1992), subjects tend to be blind to a visual stimulus presented briefly after another stimulus when the first stimulus is consciously perceived. This phenomenon arises because of the limited availability of attentional resources: the brain has difficulty processing the second of the two images presented in rapid succession since it is processing of the first of the two stimuli. However, open-monitoring/mindfulness type meditation, by disengaging some of the brain resources and leading to a more efficient cognitive processing state, has been shown to decrease the magnitude of the attentional blink effect (Slagter et al., 2007; Van Leeuwen et al., 2009).

The Stroop task (Stroop, 1935) uses color names written in congruent and incongruent ink color. Participants are asked to indicate the color of the ink verbally or by pressing a key. When the ink color and the word name mismatch (incongruent case), reaction time to indicate the ink color is slowed down (and accuracy decreased) due to involuntary automated processing of the conflicting semantic contents of the stimuli, relative to the congruent stimulus. This effect is termed the Stroop interference effect and the paradigm thus mobilizes executive attention and conflict monitoring capacities. It has been previously demonstrated that decreases in Stroop interference are seen in long-term meditators with experience in both focused attention and open-monitoring practice typical of Buddhist training regimens (Chan and Woollacott, 2007; Moore and Malinowski, 2009; Teper and Inzlicht, 2013) as well related to meditation interventions including both mindfulness and Transcendental Meditation (Alexander et al., 1989; Wenk-Sormaz, 2005; Anderson et al., 2007) and these improvements have been conceptualized as improvements in executive attention and cognitive flexibility and decreases in automatic responding patterns. We predicted that subjects would perform better after the 3-month retreat related to enhanced cognitive flexibility and decreased automated interference from semantic processes.

Finally, the global-local task is a measure of the dispositional focus of attention at either a global or more local level and the ability to switch between these levels of visual attention. This test is based on the fact that due to the limited attentional processing capacities of the brain, when attending to the global shape of an object less attention is available to attend to the local details and vice versa. Moreover, information in the non-attended level of processing interferes with the attended level of processing due to involuntary capture of attention. Work by Navon (1977) and replicated by others since has found that most people spontaneously show a bias toward the global level of perception indicated by both (1) faster responses to a task to identify the global letter than to identify the local letters when the two letters are incongruent and, (2) greater global biasing on the local task relative to local biasing on the global task. On the local task (instructing press button corresponding to the local letters) when the global letter is incongruent vs. congruent with the local letters there is an increased reaction time/decreased accuracy related to global-biasing. Conversely, on the global task (instructing press button corresponding to the global letter), when the local letters are incongruent vs. congruent with the global letter there is an increase in reaction time/decreased accuracy related to local biasing. There has been limited previous experimental work with the global-local task in relation to meditation one such study has found improvements in Stroop performance in participants with experience in a variety of Buddhist meditation practices relative to controls but no changes on global-local task performance (Chan and Woollacott, 2007). Another study has found that Zen meditators with a predominance of training in both focused attention and open-monitoring practice demonstrate faster reaction times across all stimuli and a decrease in the magnitude of global attentional bias relative to controls (Van Leeuwen et al., 2012). In contrast a group of practitioners with primary experience in focused attention practice alone tended to show local attentional bias instead of the normal global attentional bias, and a 4 day intensive retreat in open-monitoring practice tended to reduce the magnitude of this local attentional biasing such that the reaction times to the two stimuli were more equivalent. These investigators interpreted this set of findings as indicative of the fact that focused attention practices may tend to lead toward local feature biasing whereas open-monitoring practices may lead toward a reduction in biasing to either the local or global levels (Van Leeuwen et al., 2012).

A recent study showed that increased local attention bias during the global task was correlated with an increased magnitude of the attentional blink and that greater global attention bias overall during this task predicted decreased attentional blink consistent with the notion that diffusion of attention may be indexed by both greater relative global compared to local attentional bias and decreased attentional blink magnitude (Dale and Arnell, 2010).

Our hypothesis was that at the end of the retreat our subjects would be able to better redirect their attention than at the beginning (increased attentional flexibility) and would thus show a decrease specifically in the magnitude of the local biasing effect during the global task and global biasing effect on the local task. This was hypothesized based on the fact that while some aspects of the Isha Yoga meditation practices (*Lingasanchalana*, *Samyama*) have a focused attention quality to them, there is also an open-monitoring aspect to the *Shoonya* practice that might lead to a decrease in attentional biasing.

## 2. Methods

### 2.1. Participants

Subjects were tested at the beginning and at the end of a 3-month retreat in the Isha center in Nashville, TN, USA. 103 Isha Yoga meditators participated in the study. All participants signed consent forms and the study was approved by the Quorum independent ethical review committee. 89 of the 103 participants tested at the beginning of the study completed the retreat and were recorded again at the end of the retreat. Seven participants did not understand the tasks correctly despite the training session and were removed from analysis. Thus, results presented here comprise 82 participants (mean age: 37.5 years old; max: 63; min: 21, ± 9.4).

### 2.2. Meditation practice prior to the retreat

Prior to the retreat the participants had practiced *Shoonya* meditation during an average of 4 years (± 2.8 years), 6.5 days a week (± 0.5 days). Although we do not have access to data concerning the precise time spent practicing *Shoonya* meditation every day, the Isha Yoga school recommends practicing it at least 15 min twice daily. Only 50 participants practiced *Samyama* meditation prior to the retreat, and they practiced it for an average of 3 years (± 2 years), in average 3.2 days per week (± 2.1 day). No participant practiced *Lingasanchalana* meditation prior to the retreat.

### 2.3. Description of the practices during the retreat

The 3 month retreat required participants to engage in daily meditative practices. For the first 6 weeks of the retreat all participants practiced *Samyama* meditation for 30-50 min and *Shoonya* meditation for 30 min daily. For the last 6 weeks, they practiced *Lingasanchalana* meditation for 2–3 h every day. Beside pure meditative practices participants also practiced yoga postures (2 h every day) as well as physical exercise (40 min every day) and chanting (1 h every day). Finally during the first 6 weeks of the retreat only they practiced breath watching (1 h a day) and specific body postures called kriyas (2 h a day).

### 2.4. Protocole

#### 2.4.1. Recording schedule

At the beginning of the retreat, participants were tested on all three tasks and they were again tested on the same three tasks at the end of the retreat. Due to the relatively large number of subjects participating in the study, the pre- and post-tests could not all be done on the same day. Days of recording for pre-test started from the first day and finished up to 12 days after the retreat started. Recording for the post-tests started 16 days before and finished 3 days after the end of the retreat. We used the number of days relative to the beginning and to the end of the retreat as a statistical regressor to assess if this could have influenced our results (see Statistics).

#### 2.4.2. Task presentation

For all stimulus presentations, we used desktop computers running the Matlab Psychophysics toolbox (v3.0.8) under Windows XP operating systems. Stimuli were presented on a 17” DELL M781 mm CRT computer screen. The computer screen was set to 120 Hz and a resolution of 800 × 600. Two identical computers and screens were used to collect the data. Subjects sat 50 cm from the screen. Before performing the experiment, for training purposes, participants performed a shorter version of each of the tasks (three trials of the attentional blink task, 12 trials of the Stroop task and four trials of the global-local task). During this training phase the participants were allowed to repeat the training tasks again if they had problems understanding them.

### 2.5. Stroop task

**Figure 1:**
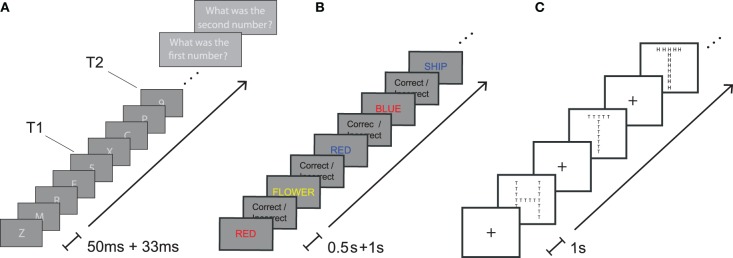
**Task paradigms**. **(A)** Attentional blink **(B)** Stroop task **(C)** Global-local task.

The Stroop task consisted of showing the capitalized words “RED,” “BLUE” and “YELLOW,” or the control words “LOT” (control word for “RED”), “SHIP” (control word for “BLUE”) and “FLOWER” (control word for “YELLOW”) on a computer screen (Raz et al. 2007). Three sequences of 60 stimuli each were presented to subjects. Each sequence started with the text “Press the key matching the COLOR OF THE INK as fast as possible - Please use ‘Z’ for red, ‘X’ for yellow, and ‘C’ for blue - Press enter to start.” Subjects were able to rest from 1 to 5 min between sequences. Each sequence of 60 stimuli contained 12 unique stimuli repeated 5 times. Three of the 12 stimuli were the color word with matching ink color. Three of the 12 stimuli were the control words and six of the stimuli were the color words with non matching ink color - Stroop condition. The stimulus sequence was pseudorandomized such that no consecutive stimuli had the same color. The ‘Z,’ ‘X,’ and ‘C’ keys had a small sticker indicating the color they corresponded to. The color was indicated with words (“RED,” “BLUE,” “YELLOW”). This was done to make sure that subjects were not confused during the experiment about which button corresponded to which color. The computer screen background was gray (#808080 - at the midpoint between black and white in the RGB color scale). We used the standard red, blue and yellow inks in the RGB color scale (#FF0000, #0000FF, and #00FFFF respectively) using Arial font and letter size of 9 mm. Stimulus presentation and keystroke latency acquisition were performed using the Matlab psychophysics toolbox (Brainard, 1997). The timing of each trial was as follows: each word was presented until the subject pressed one of the three keys. The word was immediately followed by a feedback word “Correct” or “Incorrect” in black ink and using Arial font and letter size of 4.5 mm based on whether the subject had provided a correct or an incorrect response. The feedback word remained on the screen for 0.5 s. Then a 1-s gray screen was presented until the next trial. Subjects performed 3 runs of 60 stimulus presentations (Figure 1B).

### 2.6. Attentional blink task

In the attentional blink task, series of letters were rapidly presented to subjects (Figure 1A). These series of letters contained one or two numbers that subjects were subsequently asked to report. To avoid potential ambiguities between letters and numbers, we used only 21 letters (A, C, D, E, F, G, H, J, K, L, M, N, P, R, T, U, V, W, X, Y, Z) and 8 numbers (2, 3, 4, 5, 6, 7, 8, 9). The sequence length was fixed to 19. Each letter or number was presented in white (gray level 255) on a gray background (gray level 168) for exactly 50 ms followed by 33 ms of a gray screen. The size of the letter was about 2 cm on the screen corresponding to a visual angle of 2.3 degree. A first number T1 was presented at a position chosen randomly between 3 and 11 in the sequence (Slagter et al., 2007). On one third of the trials, no second number was presented (condition T2 absent). On one third of the trials, a second number was presented three letters after the first number (condition T2 short; for example, if the first number was at position 10, the second one was at position 14). At this position (332 ms post T1) the attentional blink effect is expected to be maximal. Finally, on one third of the trials, a second number was presented seven letters after the first number (condition T2 long), 664 ms post T1 which is outside the significant attentional blink time window. After each sequence, participants were asked to enter the first and the second number (0 if they thought there was none) and then confirm their choice before the next trial. Participants were instructed to guess the numbers if they thought a second number was present but were not sure what it was. Subjects were tested on a total of 120 trials. Stimuli presentation latency and screen vertical blanking were carefully monitored using custom Matlab scripts and CRT analog computer monitors were used to ensure that the presentation time on the screen matched the presentation time in the experimental script.

### 2.7. Global-local task

In the global-local task, subjects were shown large letters (H and T) on a computer screen. The large letters were made up of an aggregate of small letters that could be congruent (large H made of small Hs or large T made of small Ts) or incongruent (large H made of small Ts or large T made of small Hs) with respect to the large letter. The small letters were 0.8 cm high and the large letters were 8 cm high on the computer screen. A fixation cross was present at all times except when the letters were presented. Letters were shown on the computer screen until the subject responded. After each subjects response, there was a delay of 1 s before the next stimulus was presented. Before each sequence of letters, instruction were shown on a computer screen indicating to subjects whether they should respond to the presence of small (local condition) or large (global condition) letters. We instructed subjects to categorize specifically large letters or small letters and to press the letter H or T on the computer keyboard to indicate their choice. Subject performed a total of 52 trials in 4 sessions of 13 trials each. In sessions 1 and 3, subjects were instructed to focus on large letters and in session 2 and 4 they were instructed to focus on small letters (Figure 1C).

### 2.8. Statistics

Since our data did not fit the normal distribution (Kolmogorov–Smirnov test), we used non-parametric statistic Wilcoxon ranksum tests and used Bonferroni correction for multiple comparisons. For all tests, the degree of freedom was 81. For assessing the effect that meditation experience and age had on results, we used a general linear model and bootstrap statistics. We used Matlab to perform all statistical tests.

## 3. Results

### 3.1. Stroop task

#### 3.1.1. Accuracy

**Figure 2:**
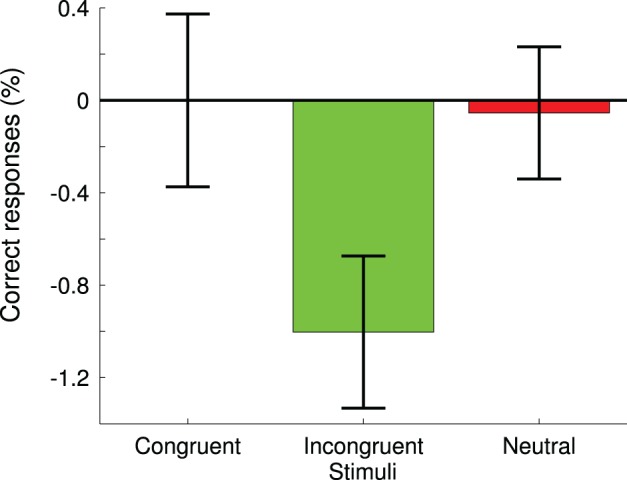
**Differences of pre- vs. post-retreat test mean correct responses to the Stroop task for all types of stimuli**. Standard error of the mean are shown for each condition.

In the Stroop task, subjects tended to commit less errors to incongruent stimuli at the end of the retreat (*p* < 0.05, 86.5% correct responses at pre-test vs. 87.4% correct response at post-test) (Figure 2). We found no significant difference in accuracy to congruent (*p* = 0.9) or neutral (*p* = 0.7) stimuli between pre- and post-retreat tests. For each subject, we also calculated the Stroop Interference (SI) by subtracting the accuracy score to incongruent stimuli from accuracy score to congruent stimuli. We found a significant diminution of the Stroop Interference after the retreat compared to before the retreat (*p* < 0.05, mean SI at pre-retreat test was 1.8, mean SI at post-retreat test was 0.8).

#### 3.1.2. Reaction time

We did not observe any significant effects in reaction time for any of the comparisons we tested. Specifically, we did not observe a difference in RT for incongruent, congruent, or neutral stimuli when comparing pre- and post-retreat assessments. Similarly, we did not observe a significant difference in the RT Stroop Interference, computed as the difference of RT for congruent and incongruent stimuli for each subject, between pre- and post-retreat assessments.

### 3.2. Attentional blink task

**Figure 3:**
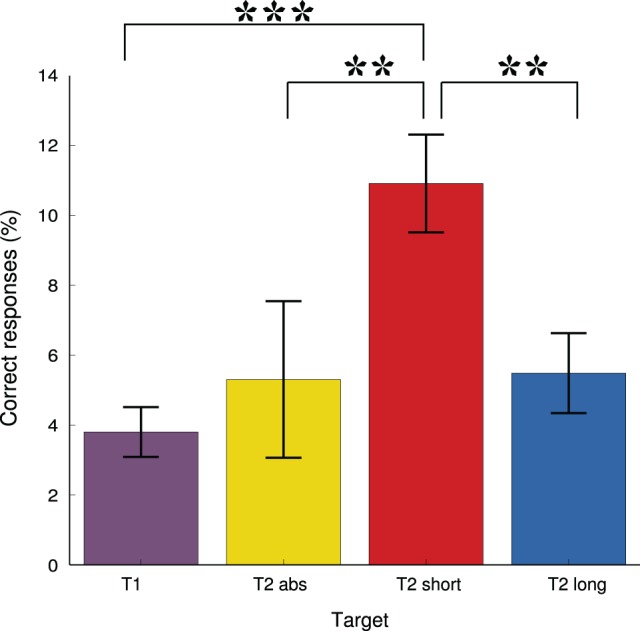
**Mean accuracy improvement for each target type between pre and post test in the attentional blink task**. Standard error of the mean are shown for each condition. ^**^indicates *p* < 0.005 and ^***^indicates *p* < 0.0005.

Attentional blink performances were improved at the end of the retreat when compared to performances at the beginning: the rate of detection of the T2 target within the window of the attentional blink (T2 short; target T2 following a short interval after T1) was 59% at pre-test vs. 70% at post-test (*p* < 0.0001). Significant increases in performance between pre- and post-tests were also found for the targets T1, and T2 long (*p* < 0.05; mean detection rate of T1 at pre-test 84 vs. 87% at post-test; mean detection rate of T2 long at pre-test is 75 vs. 81% at post-test). No significant difference for detection of the absence of T2 (T2 absent) was found (mean T2 absent detection rate at pre-test 55 vs. 61% at post test). However, as shown in Figure 3, comparison of post-test improvement between each type of stimulus revealed that performances increased significantly more for detection of T2 presented in a short interval than for any of the other targets (T2 short performance increase vs. T1 performance increase *p* < 0.0002; T2 short performance increase vs. T2 long performance increase *p* < 0.001; T2 short performance increase vs. T2 absent performance increase *p* < 0.005).

To assess potential short term learning effects, we divided subjects trials into 2 groups of equal sizes: the first half and the second half of the trials. Within each pre and post test session, for each type of target, we did not find any significant differences in performances between the first half and the second half of the trials (ranksum test).

### 3.3. Global-local letter task

#### 3.3.1. Accuracy

**Figure 4:**
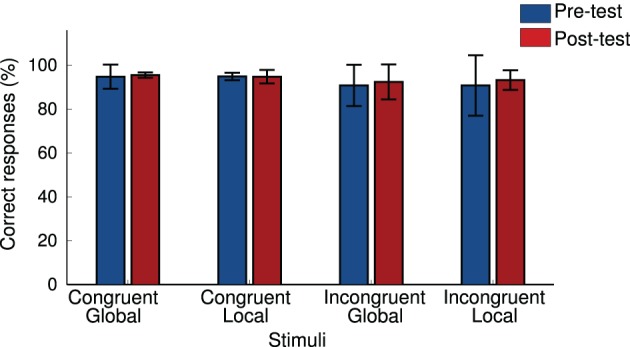
**Mean correct responses to the local and the global task in congruent and incongruent condition at pre-test and post-test**.

At the beginning and at the end of the retreat, participants made significantly less errors on congruent than on incongruent trials during both the global and the local tasks (*p* < 0.001) see Figure 4. There were no significant differences in performance between pre- and post-retreat tests in either the congruent or incongruent conditions for the local (congruent condition *p* = 0.6; incongruent condition *p* = 0.2) or global (congruent condition *p* = 0.7; incongruent condition *p* = 0.6) tasks. We computed the congruency effect interference score (performance on congruent minus performance on incongruent trials) but did not find significant differences between pre- and post- retreat scores on this measure either (local task: *p* = 0.7; global task: *p* = 0.1).

#### 3.3.2. Reaction times

**Figure 5:**
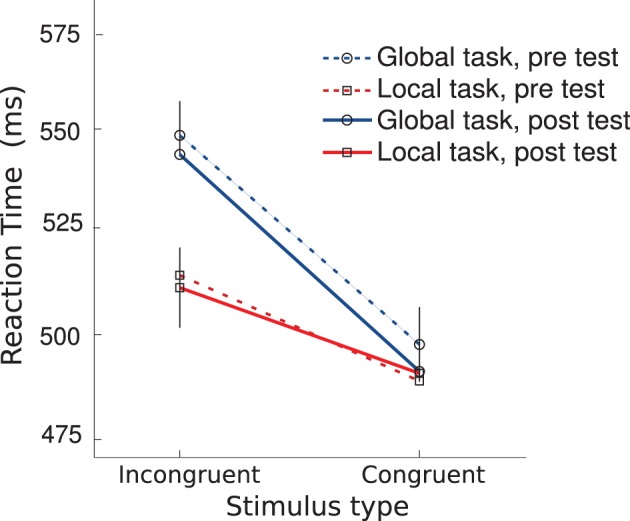
**Mean reaction time to respond to global and local features at pre and post test depending on the congruency of the stimuli in the global-local task**.

Participants were significantly faster in responding to congruent than incongruent trials during the global task both before and after the retreat (*p* < 0.005; see Figure 5) but we did not find a significant congruency effect for the local task, although we did observe a trend (*p* = 0.057). We found no significant differences between RTs before and RTs after the retreat for any of the specific trial types as a class. However, there was a significant faster mean RT in response to incongruent stimuli in the local versus global task (*p* = 0.04) before the retreat, and only a trend difference (*p* = 0.1) after the retreat.

### 3.4. Attentional blink and global-local performance

We tested the hypothesis that performances in the global-local task would be correlated with performance in the attentional blink task. We divided participants into quartiles based on their scores on the local-interference (defined as the difference between RTs to incongruent and congruent conditions when subjects had to respond based on the global stimuli) and global interference (defined as the difference between RTs to incongruent and congruent conditions when subjects had to respond based the local stimuli) measures, for both pre and post retreat assessments. Low local interference participants had scores within the first quartile (pre retreat, *n* = 20; post retreat, *n* = 21) and high local interference participants had score within the last quartile (pre retreat, *n* = 21; post retreat, *n* = 21). We used linear regression to assess any relationship between attentional blink performances and local interference scores and did not find any significant correlations either before or after the retreat. We did the same analysis using the global-interference measure and similarly found no significant correlations.

### 3.5. Influence of age and prior meditation experience on task performances

We used a general linear model to assess the effect of participants age and prior meditation experience on task performances. For each task, we computed the correlation between years of *Shoonya* meditation practice (since it was the only meditation practice shared by all participants prior to the retreat, see section 2.2), age of the participants, and performance for each of the tasks for the pre-retreat assessments. In the attentional blink task, age and previous *Shoonya* meditation experience of the participants both had a significant negative influence on T1 response accuracy (age: regression coefficient (reg. coef.) = −0.46, *p* < 0.005; previous meditation experience: reg. coef. = −0.04; *p* < 0.005) T2 long (age: reg. coef. = −0.22, *p* < 0.01; previous meditation experience: reg. coef. = −0.30; *p* < 0.01) and T2 short response accuracy (age: reg. coef. = −0.29, *p* < 0.01; previous meditation experience: reg. coef. = −0.14; *p* < 0.01 ) but not T2 absent (*p* < 0.1) response accuracy. In the Stroop task, the age and meditation experience of participants did not affect reaction time. However, we observed a significant positive influence of these factors on accuracy to both incongruent (age: reg. coef. = 0.11, *p* < 0.005; previous meditation experience: reg. coef. = 0.16; *p* < 0.005) and neutral stimuli (age: reg. coef. = 0.03, *p* < 0.05; previous meditation experience:reg. coef. = 0.05; *p* < 0.05). Both increased age and prior meditation experience significantly correlated with decreases of Stroop interference (age: reg. coef. = −0.10, *p* < 0.01; previous meditation experience: reg. coef. = −0.07; *p* < 0.01) Finally, there was no significant influence of either age or meditation experience on performance to congruent stimuli. In the global-local task, we did not observe any significant effect of age or meditation experience on accuracy or RT to either type of stimuli.

## 4. Discussion

We tested 82 meditation practitioners at the beginning and at the end of a 3 month full time meditation retreat using three attentional tasks: an attentional blink task, a Stroop task and a global-local task. Our results show a modest decrease in the magnitude of the attentional blink at the end of the retreat compared to the beginning. Unexpectedly, we also found that previous meditation experience correlated with decreased performance in accurately recognizing both the T1 and T2 stimuli of the attentional blink paradigm. Comparing the magnitude of improved performance on the attentional blink paradigm to previous studies indicated a similar magnitude of improvement in a control cohort of novices with minimal meditation experience and no significant intervention. This is thus casting doubt on the interpretation that this effect was a specific result of meditation during the retreat and more in support of this being related to improved performance secondary to simple learning mechanisms. Comparing pre- and post-retreat assessment of conflict monitoring and automated responding as assessed by the Stroop paradigm we observed decreased Stroop interference related to improved accuracy on the incongruent trials of the Stroop task. Congruent with this, previous meditation experience in our cohort also correlated with increased accuracy on incongruent trials and decreased Stroop interference at the baseline pre-retreat assessment. We found no significant main effects of the retreat on accuracy or reaction time in the incongruent trials of the global-local task although a pattern of relatively faster reaction times on the local task relative to the global task both pre and post retreat indicated that this cohort showed an unusual local attentional biasing, possibly related to the practices in this tradition being more in the focused attention domain.

### 4.1. Effect of recording schedule

Because of the large number of subjects and our limited data collection capabilities, some subjects were recorded after the beginning of the retreat and some subjects were recorded before the end of the retreat. We thus checked for correlation of pre- and post test performances with pre- and post- test date expressed as the day the test occurred relative to the beginning and the end of the retreat. Overall, we computed 18 regressions scores that did not lead to significant correlation with pre- or post-retreat test date after applying the Bonferroni correction for multiple comparisons.

### 4.2. Isha yoga meditation and reduction of stroop interference

Our results show increased accuracy scores on incongruent Stroop stimuli at the end of the retreat compared to the beginning. In addition, previous meditation experience correlated with more accurate performance at the baseline assessment. These results are in accordance with a previous reports demonstrating less Stroop interference in experienced Buddhist meditators compared to controls (Moore and Malinowski, 2009; Teper and Inzlicht, 2013) as well as in a cohort of diverse long term meditators of different traditions (Chan and Woollacott, 2007). Moreover, decreased Stroop interference after training in mindfulness meditation in college-age students (Wenk-Sormaz, 2005), as well as after training in Transcendental Meditation in older adults (Alexander et al., 1989) have been previously reported, although one report found no changes in Stroop task performance after a mindfulness-based stress reduction (MBSR) intervention (Anderson et al., 2007). The decrease in Stroop interference we report here is consistent with the notion that the flexible executive attention and reduction in automatic responding measured through the Stroop color-word interference task is one of the domains of attention positively affected through this meditative practice.

It is of note that a recent study utilizing EEG concomitantly with Stroop performance (Teper and Inzlicht, 2013) found that, in addition to decreased Stroop interference, experienced meditators showed increased amplitude of the ERN event-related potential known to be related to the engagement of anterior cingulate cortex in conflict monitoring, thus providing some indirect evidence that these Stroop findings may be related to enhanced anterior cingulate functioning related to the long hours of practice during meditation retreat.

### 4.3. Meditation and reduction of the attentional blink

We observed an increased detection rate (70%) of the target T2 presented in the short interval after T1 (window of the attentional blink) relative to the pre-retreat test (59%). One previous study assessed the effect of meditation and the attentional blink effect.

Slagter et al. (2007) did a pre/post testing before and after a 3 month intensive Vipassana (open-monitoring/mindfulness) meditation retreat with 17 expert meditation practitioners of diverse meditation traditions compared to a group of novice meditators.

Our study found an improvement in the meditation retreat participants equal to the control group participants in the Slagter study (from about 60 to 70% T2-short correct) and not as robust as the meditator group participants in that study (who showed an average of about 60 to 80% T2-short correct). As we did not have a control group to compare the meditator cohort data in this study, we cannot assume that the rather modest improvement in performance was specifically related to the intensive meditation practice during the Isha Yoga retreat. Of note, during the Isha Yoga meditation retreat considered here, participants spent about 1.5–4 h daily sitting in silent meditation whereas in Slagter et al. (2007)'s study open-monitoring Vipassana meditation during the retreat was practiced intensively 10–12 h a day. It is possible that with more intensive practice of sitting meditation the observed effects of Isha Yoga meditation practice would have been more robusts.

The contrast in findings between this study of the effects of the Isha Yoga 3-month retreat and the earlier report before and after a 3-month Vipassana retreat seems to indicate that intensive open-monitoring/mindfulness training may more specifically modulate the attentional capacities underlying the performance of the attentional blink paradigm. This is possibly related to Vipassana practice engaging the attentional mechanisms on present moment awareness with a greater focus on sensitively attending to internal and external experience with concomitant purposeful cognitive non-elaboration on experience. In contrast, the *Shoonya*, *Samyama*, and *Lingasanchalana* practices done in the Isha Yoga retreat have a less explicit focus on open-monitoring and are instead favorising the focused attention spectrum of practice. Consistent with this interpretation, in contrast to the negative correlation we obtained between previous Isha Yoga meditation practice and attentional blink performance at baseline, Van Leeuwen et al. (2009) showed that older adult expert meditation practitioners with extensive experience in both focused attention and open-monitoring practice did not show the same decrease in attentional blink performance relative to young adults as older non-meditators, seeming to implicate more pure open-monitoring practice as important to improving attentional blink performance.

Although our modest improvement in performance and lack of a control cohort precludes our attributing the improvements that we did observe in the attentional blink paradigm to the meditation practiced in retreat, it is also possible that some of this improvement may have been due to the “open monitoring” aspect of the diverse Isha Yoga meditation practices. Indeed, Olivers and Nieuwenhuis (2005, 2006) demonstrated that the attentional blink can be attenuated if instead of concentrating hard on the task, the subject is asked to simultaneously perform an additional task or even simply instructed to concentrate less on the task. It is possible that at the end of the retreat our subjects might have engaged a more diffuse attentional deployment, preventing them from allocating too much attentional resource to the initial T1 stimulus in the stream of stimuli.

Overall we take the smaller improvement in attentional blink performance in this Isha Yoga retreat cohort relative to the previous Vipassana retreat cohort assessed by Slagter et al. (2007) using a very similar attentional blink paradigm as likely related to the difference in attentional deployment in more pure open-monitoring practice resulting in greater diffusion of attention and resultant optimization of attentional resources on this task assessing temporal attention capacities. Indeed, the fact that meditation experience in the Isha Yoga cohort actually correlated with worse attentional blink performance at the pre-retreat assessment tends to argue that this form of meditation practice did not specifically lead to improved attentional blink performance.

### 4.4. Global-local task

Both at the beginning and at the end of the retreat, participants were faster to respond in the local task than in the global task to stimuli with incongruent global and local features. Moreover, participants were faster to respond at the global level when stimuli were congruent than when stimuli were incongruent and the speed of responding was thus biased by local features. However, there was only a trend difference in reaction time between congruent and incongruent stimuli when responding to the local level at both the beginning and end of the retreat, indicating a decreased magnitude of global biasing relative to local biasing. These results suggest that overall this global-local paradigm tended to evoke in this cohort a non-standard tendency for local processing to predominate over global processing. Previous literature (Navon, 1977) has demonstrated that most individuals show a global precedence effect and thus respond more quickly on a global compared to a local task when shown stimuli with conflicting local and global features. Thus it seems likely that this cohort of Isha Yoga meditation practitioners shows a greater tendency toward local processing, which may be related to their meditation practice. In particular, given previous reports of focused attention practices leading to greater local processing precedence (Van Leeuwen et al., 2012) this finding seems to further support that Isha Yoga meditation practices are primarily concentrative in nature.

The only pre-/post- differences in performance on the global-local task found was related to slight decrease in the magnitude of the faster reaction time to incongruent stimuli in the local relative to global task condition. This finding indicates a possible slight relative increase in global attentional processing after the retreat, somewhat in line with the hypothesized increase in global attentional biasing that we predicted might accompany improvements in attentional blink performance. However this metric is not as specific and robust a metric of global processing as the global-local interference effects which were not found to be affected by the retreat so reading much into this finding is unwarranted.

It is of note that the one previous study assessed the global-local task as well as Stroop performance in experienced meditators vs. controls also found improvements in Stroop task performance but no effect of meditation in performance on the global-local task (Chan and Woollacott, 2007). Although we did not replicate the results of Dale and Arnell (2010) showing a correlation between greater attention to the local level in the global-local Navon task and greater magnitude of the attentional blink, we did demonstrate the complementary finding that meditators in this tradition of more focused attention practices show biasing toward the local processing on the Navon task and those with greater pre-retreat practice also tended to display greater attentional blink amplitude.

Another explanation pertains to the type of meditation we studied. Other types of meditation involving visualization such as those practiced in Tibetan Buddhism might be expected to engage a more robust effect compared to Isha Yoga meditation which does not involve any visualization. For example, Carter et al. (2005) have found higher performance in the maintenance of bistable visual stimuli in Tibetan Buddhist meditators practicing meditation requiring to focus on one point (real or imagined), compared to the same meditators practicing compassion meditation, consistent with the now well established notion that different meditation practices engage attentional processes in very different ways and thus have different effects on many aspects of the attentional system.

## 5. Conclusion

Our study reinforces that increased cognitive flexibility and decreases in automatic responding as indexed by the Stroop task is one likely concomitant to diverse forms of meditative practice including Isha Yoga practices which involve more focused attentional mechanisms as well as some explicit focus on self-transcending. The results point toward a negligible effect of this form of more focused meditation on the efficiency of temporal attentional resource allocation indexed by the performance on the attentional blink task in contrast to the improvements previously observed with open-monitoring practices. Lastly, there is some indication that this form of practice may predispose individuals toward greater local precedence in spatial visual attention instead of the more typical global precedence seen in the population through performance on the Local-Global task. The Stroop task result is the most robust since increases in accuracy in the task seen between pre- and post- recording is reinforced in the observed correlation between previous meditation experience and improved performance on this task. All of these effects would be much more reliably interpreted in the presence of a control group, which is the primary limitation of this study.

### Conflict of interest statement

The authors declare that the research was conducted in the absence of any commercial or financial relationships that could be construed as a potential conflict of interest.
